# Magnetic Flux Sensor Based on Spiking Neurons with Josephson Junctions

**DOI:** 10.3390/s24072367

**Published:** 2024-04-08

**Authors:** Timur Karimov, Valerii Ostrovskii, Vyacheslav Rybin, Olga Druzhina, Georgii Kolev, Denis Butusov

**Affiliations:** 1 Youth Research Institute, Saint Petersburg Electrotechnical University “LETI”, 197022 Saint Petersburg, Russia; tikarimov@etu.ru (T.K.); vyostrovskii@etu.ru (V.O.); 2 Computer-Aided Design Department, Saint Petersburg Electrotechnical University “LETI”, 5 Professora Popova St., 197022 Saint Petersburg, Russia; vgrybin@etu.ru (V.R.); osdruzhina@etu.ru (O.D.); gyukolev@etu.ru (G.K.)

**Keywords:** Josephson junction, artificial neuron, magnetic flux sensor, spiking neural network, superconductive device

## Abstract

Josephson junctions (JJs) are superconductor-based devices used to build highly sensitive magnetic flux sensors called superconducting quantum interference devices (SQUIDs). These sensors may vary in design, being the radio frequency (RF) SQUID, direct current (DC) SQUID, and hybrid, such as D-SQUID. In addition, recently many of JJ’s applications were found in spiking models of neurons exhibiting nearly biological behavior. In this study, we propose and investigate a new circuit model of a sensory neuron based on DC SQUID as part of the circuit. The dependence of the dynamics of the designed model on the external magnetic flux is demonstrated. The design of the circuit and derivation of the corresponding differential equations that describe the dynamics of the system are given. Numerical simulation is used for experimental evaluation. The experimental results confirm the applicability and good performance of the proposed magnetic-flux-sensitive neuron concept: the considered device can encode the magnetic flux in the form of neuronal dynamics with the linear section. Furthermore, some complex behavior was discovered in the model, namely the intermittent chaotic spiking and plateau bursting. The proposed design can be efficiently applied to developing the interfaces between circuitry and spiking neural networks. However, it should be noted that the proposed neuron design shares the main limitation of all the superconductor-based technologies, i.e., the need for a cryogenic and shielding system.

## 1. Introduction

Sharing the quantum properties of a superconducting state, the systems with Josephson junctions (JJ) [1] enable various applications in high-performance computing and advanced sensing. Rapid single flux quantum (RSFQ) circuitry is a well-developed fabrication technology, which makes use of magnetic flux quanta produced by JJs to represent digital information carried by picosecond pulses via superconducting lines. Compared to semiconductor logic circuits, RSFQ electronics benefit from low power consumption and unprecedented clock rates. Numerous ultrafast circuits were created employing the last property, such as rapid single flux quantum digital dividers that operate up to 770 GHz [2], digital signal processors with 20 to 40 GHz clock rates [3,4], and serial microprocessors with nearly 20 GHz operating frequencies [5]. Among JJ-based technologies, superconducting quantum interference devices (SQUID) merit particular interest. These devices utilize a special JJ-based superconducting circuit to detect very weak magnetic fields, thereby SQUID-magnetometers are widely used in biology and medicine, e.g., for magnetic resonance imaging [6], magnetoencephalography [7], and scanning microscopes [8,9]. Classic SQUID types include radio frequency (RF) and direct current (DC) SQUIDs; on their basis, hybrids can be made, e.g., D-SQUID [10,11]. One of the most popular commercial applications of SQUIDs is magnetic property measurement systems [12].

As the current silicon transistor-based processors approach the limits of miniaturization, research on non-von Neumann architectures for in-memory computing and machine learning intensifies significantly. Neuro-inspired computing electronics, which incorporate neurobiological features, may also offer an energy-efficient solution for artificial intelligence workloads [13]. Memristive systems are typically viewed as the fundamental circuit elements for such solutions, serving both for implementing the mechanisms of spike generation in neurons [14] and for representing the synaptic plasticity [15]. Nanoscale thin film (metal-insulator-metal) resistive switching devices were introduced as memristors only in 2008 [16]. Being compatible with complementary metal-oxide-semiconductor (CMOS) technology [17,18] is the main advantage of this type of device, while a major drawback is the large device-to-device and cycle-to-cycle variability [19]. In turn, the early mention of a JJ as a memristor dates back to 1974 [20]. Superconducting memristors [21] have the benefits of lower characteristic times (picoseconds [22] versus 10 nanoseconds [23]), lower power consumption ($10^{-17}$ J/spike for superconducting neurons [22] versus $6.3\times 10^{-15}$ J/spike for CMOS neurons [24], as a reference $10^{-11}$ J/spike for biological neurons), and greater stability, which comes at a price of cryogenic equipment. Thus, neuromorphic electronic devices are developing in parallel through competing CMOS and superconducting technologies.

Let us briefly overview the recent progress in developing JJ-based neuromorphic systems. In 2006, S.K. Dana et al. [25] reported the numerical simulation results on the neuron-like spiking and bursting activity in a resistive-capacitive-inductive shunted junction (RCLSJ) model. In 2010, P. Crotty et al. [26] presented the JJ neuron, which models the voltage dependence of typical ionic currents in biological neurons by junctions whose dynamics are governed by familiar second-order differential equations. This study also demonstrated such important characteristics of the JJ neuron as the firing threshold and refractory period. Then, in [22], the potential of the JJ neuron circuitry was experimentally demonstrated to simulate somas, axons, and adjustable synapses, as well as to perform the detection of activity states. In [27], T. Hongray et al. reported the bursting behavior produced in a system of two resistively coupled resistive-capacitive shunted junction (RCSJ) models, also explored the parameter space for various operating modes of the system. In 2016, S.E. Russek et al. [28] presented magnetically tunable JJ for synaptic constituent in neuromorphic computing. In 2019, R. Cheng et al. [29] presented neuromorphic computing circuitry components (neurons and synapses) based on quantum phase-slip and magnetic Josephson junctions. M.L. Schneider et al., in [30], proposed SQUID synapse based on the nanotextured magnetic JJs, modeled by the modified version of the RCSJ, and in [31], they provided the results for the fabricated devices. In [32], F. Feldhoff and H. Töpfer proposed the RSFQ circuit design of a spiking neural network grown around a Josephson comparator. A.E. Schegolev et al. [33] studied two superconducting JJ models of a biological neuron (from [26]) by marking areas of different operating modes on parametric planes of the corresponding dynamical systems, and suggested the circuitry for synaptic connection of the JJ neurons. The physical implementation of two- and three-junction superconducting quantum interferometers with Josephson weak links based on gold nanowires are investigated in [34]. Another study [35] described a family of logic/memory cells in which stored multi-bit data are encoded by quasi-analog currents or magnetic flux in superconductor loops, while transmitted data are encoded as the rate of SFQ pulses. Ref. [36] proposed a technique to improve artificial neural network (ANN) performance by increasing their energy efficiency and speed of operation, and also sought to extend the utility of ANNs by natively adding functionality of spiking operation.

Besides JJ-based electronics, other superconducting neuromorphic technologies exist, including superconducting nanowires and optoelectronic circuits. An example of the first type is the study by E. Toomey et al. [37], where the authors compared the platform of superconducting nanowires with JJ architectures to model spiking neural networks, noting the advantages of the presented synapse design in the fan-out property. An example of research on superconducting optoelectronic circuits for neuromorphic computing is [38], where J.M. Shainline et al. proposed a hybrid hardware platform that combines semiconducting few-photon light-emitting diodes with superconducting-nanowire single-photon detectors to behave as spiking neurons.

A new paradigm of quantum machine learning includes spiking neuron models and also requires the development of neuromorphic sensor prototypes to encode detected quantities into spike signals. Neuromorphic sensors for detecting gas [39] and humidity [40] are perfect examples of applications in conventional electronics. Retinomorphic vision sensor design is another promising field of research and development [41], aiming to reproduce spiking behavior in CMOS image sensors, and impart on them a natural ability to detect events. Ref. [42] investigated the presence of unique solutions and quasi-uniform stability for a class of fractional-order uncertain BAM neural networks utilizing the Banach fixed point concept. For bio-inspired robotics, researchers have recently proposed spiking-output tactile sensors based on a piezoelectric field-effect transistor [43], epitaxial VO_2_ volatile memristor [44], and Mott NbO_x_ memristor [45]. The last sensor presents a general-purpose device acting like an afferent nerve, which transforms the voltage into a spike train (Hodgkin’s class 1 excitation).

**Figure 1:**
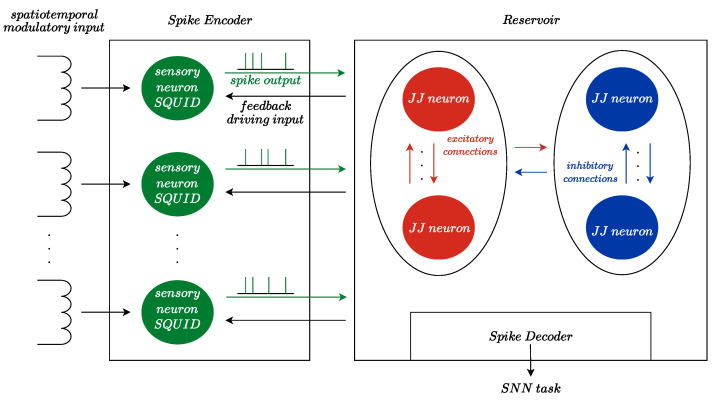
The concept of online magnetic field forecasting using reservoir SNN.

In our previous works, we presented a single-coil metal detector with information encoding using a spiking oscillator [46] and memristor-based capacitance-sensing neuron [47]. The current study proposes a novel approach by combining the DC SQUID with a JJ-based neuron. The proposed neuron model is suggested to be sensitive to an external magnetic field. The considered model comprises memristive elements, being a combination of passive R-L-C elements with Josephson junctions, which results in a structure behaving in a bio-inspired manner. Thus, the proposed scheme is a combined sensor-transducer device whose behavior can significantly vary while influenced by slight magnetic field fluctuations. In this case, the external magnetic flux acts as a modulatory input signal, influencing the rate of spike generation. The supposed application example for such a device is illustrated in Figure 1. An array of neuron-SQUIDs may constitute a spike encoder to record the spatiotemporal dynamics of the measured magnetic field for the spiking neural network (SNN). The example shows a reservoir computing architecture that includes excitatory and inhibitory neurons, as well as a spike decoder that is typically represented by the output layer of neurons. In the diagram, the sensory neurons also receive feedback driving input from the reservoir. Within this configuration, the driving and modulatory inputs may affect the spike output of sensory neurons jointly. The gain modulation of the sensory neuron in the present work can be associated with one of the most established forms of attention mechanisms: an increase in spiking rate when attention is directed into the receptive field of a neuron [48]. By introducing the attention mechanism, the SNN becomes able to selectively focus on the important information in the input. That improves the network’s performance by enhancing meaningful features and smoothening semantic segmentation boundaries of the sensory perception. In this way, incorporation of the attention mechanism into superconducting SNN would make magnetometry cognitive.

The key contributions of this paper are as follows:

1. A novel magnetic flux-sensitive neuron model based on Josephson junctions is presented;
2. Potential operational modes for the proposed model are investigated via nonlinear analysis methods;
3. The numerical simulation confirms that the developed circuit acts like a sensor of flux quantified in several quanta and is able to convert the acquired values into measurable bio-inspired dynamics.

The rest of the paper is organized as follows. In Section 2, we present DC SQUID, the 3-JJ neuron model, and our 3-JJ flux-sensitive neuron model based on the mentioned models. In Section 3, the numerical results of the simulation are presented, and the action of the model as a sensor is approved. Section 4 concludes the paper.

## 2. Materials and Methods

### 2.1. Josephson Junction and DC SQUID Basics

The Josephson junction (JJ) is usually described by two Josephson Equations [49]:

(1) $$I_{J}=I_{C}\sin\delta$$

(2) $$\dot{\delta }=\frac{2e}{\hbar }V\left(t\right)=\frac{2\pi }{\Phi_{0}}V\left(t\right)$$

where δ (often denoted as ϕ) is a phase drop at JJ, V(t) is a voltage drop, $I_{J}$ is a current flow through JJ, $I_{C}$ is a critical current, and $\Phi_{0}=h/2e$ is a magnetic flux quantum. In case of π-junction, a specific type of JJ, the first Equation (1) becomes:

$$\begin{array}{c} I_{J}=I_{C}\sin\left(\delta +\pi \right)=-I_{C}\sin\delta \end{array}$$

**Figure 2:**
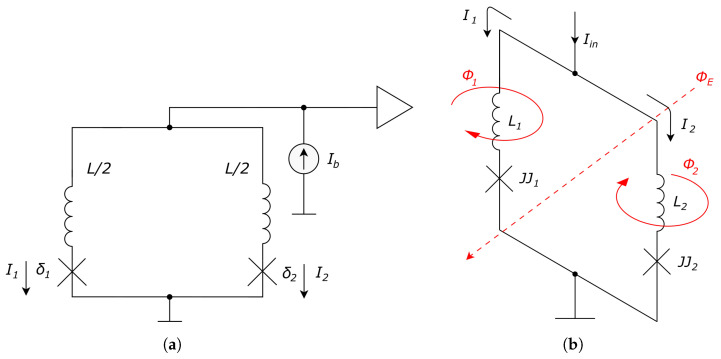
DC SQUID schematics: (**a**) electrical circuit, and (**b**) spatial scheme representing magnetic fluxes (denoted by red arrows) in the circuit, respectively.

Furthermore, we will consider only conventional JJs in this paper. Let us investigate a DC SQUID, which consists of a ring with two opposite junctions. A corresponding schematic is presented in Figure 2.

The total inductance of the ring is *L*, the flowing current is *I*, and the external magnetic flux passing through it is $\Phi_{E}$. Thus:

(3) $$\Phi_{T}=\Phi_{E}+LI$$

where the external flux is divided into two parts and flows through the left branch and right branch, with their inductance values of $L_{1}=L_{2}=L/2$. Respectively, fluxes $\Phi_{1}$ and $\Phi_{2}$ are:

$$\begin{array}{c} \Phi_{1}=L_{1}I_{1}+\Phi_{E}/2\\ \Phi_{2}=L_{2}I_{2}-\Phi_{E}/2 \end{array}$$

The difference in signs near $\Phi_{E}$ is due to opposite directions of fluxes $\Phi_{1}$ and $\Phi_{2}$. Subtracting one equation from another, one can obtain:

(4) $$\begin{array}{c} \Phi_{1}-\Phi_{2}=\Phi_{E}+L_{1}I_{1}-L_{2}I_{2}=\Phi_{E}+\frac{L}{2}\left(I_{1}-I_{2}\right) \end{array}$$

Recall Faraday’s law of induction:

(5) $$V\left(t\right)=n\dot{\Phi },$$

where *n* is the number of turns of a coil, which for DC SQUID is n=1, and V(t) is an induced voltage. Combined with the second Josephson law, it gives:

(6) $$\begin{array}{c} \frac{\Phi_{0}}{2\pi }\dot{\delta }=\Phi \to \dot{\delta }=\frac{2\pi \Phi }{\Phi_{0}} \end{array}$$

Substitute (6) into (4):

(7) $$\begin{array}{c} \frac{\Phi_{0}}{2\pi }\left(\delta_{1}-\delta_{2}\right)=\Phi_{E}+\frac{L}{2}\left(I_{1}-I_{2}\right) \end{array}$$

If the bias current $I_{b}=I_{1}+I_{2}$ flows though DC SQUID, i.e., $I_{2}=I_{b}-I_{1}$, then with $\phi_{e}=\Phi_{E}/\Phi_{0}$:

(8) $$\begin{array}{c} I_{1}=\frac{I_{b}}{2}+\frac{\Phi_{0}}{2\pi L}\left(\delta_{1}-\delta_{2}-2\pi \phi_{e}\right)\\ I_{2}=\frac{I_{b}}{2}-\frac{\Phi_{0}}{2\pi L}\left(\delta_{1}-\delta_{2}-2\pi \phi_{e}\right) \end{array}$$

The resistive-capacitive shunted junction (RCSJ) model is a classical model for continuous description of current flowing through JJ based on the first Josephson law (ideal junction), with adding parallel resistor and capacitor:

(9) $$\begin{array}{cc} I & =C\dot{V}+\frac{V}{R}+I_{C}\sin\delta \to \end{array}$$

(10) $$\begin{array}{cc} I_{1,2} & =\frac{C\Phi_{0}}{2\pi }{\ddot{\delta }}_{1,2}+\frac{\Phi_{0}}{2\pi R}{\dot{\delta }}_{1,2}+I_{C}\sin\delta_{1,2} \end{array}$$

Let us denote: $i=I/I_{C}$, $\beta_{L}=2I_{C}L/\Phi_{0}$, $\beta_{C}=I_{C}R^{2}C2\pi /\Phi_{0}$, and time scaling constant $\omega_{t}=\Phi_{0}/\left(2\pi I_{C}R\right)$, which, being applied to (10), gives the following initial value problem:

(11) $$\begin{array}{c} \left\{\begin{array}{c} \dot{\delta }=\omega_{t}x\\ \dot{x}=\omega_{t}f\left(\delta ,x\right) \end{array}\right.\to \ddot{\delta }/{\omega^{2}}_{t}=f\left(\delta ,\dot{\delta }/\omega_{t}\right) \end{array}$$

With time scaling $\tau =t\omega_{t}$:

(12) $$\left\{\begin{array}{c} \delta \tau =x\\ x\tau =f(\delta ,x) \end{array}\right.↔\left\{\begin{array}{c} \dot{\delta }=x\\ \dot{x}=f\left(\delta ,x\right) \end{array}\right.$$

Combining (8) and (12), obtain:

(13) $$\begin{array}{cc} \beta_{C}{\ddot{\delta }}_{1} & =\frac{i_{b}}{2}+\frac{\delta_{1}-\delta_{2}-2\pi \phi_{e}}{\pi \beta_{L}}-{\dot{\delta }}_{1}-\sin\delta_{1} \end{array}$$

(14) $$\begin{array}{cc} \beta_{C}{\ddot{\delta }}_{2} & =\frac{i_{b}}{2}-\frac{\delta_{1}-\delta_{2}-2\pi \phi_{e}}{\pi \beta_{L}}-{\dot{\delta }}_{2}-\sin\delta_{2} \end{array}$$

Typical parameters’ values are: $\beta_{L}=1$, $\beta_{C}=0.8$, L=10 pH, C=50 fF, $I_{C}=100\;\mu$A.

Equations that describe the dynamics of DC SQUID will be further used in our proposed flux-sensitive spiking neuron circuit.

### 2.2. Neuron Model Based on Josephson Junctions

The model of the spiking neuron based on Josephson junctions was originally proposed by Crotty et al. [26], and recently modified by Schegolev et al. [33]. The authors of the original work noticed the analogy between single-flux-quantum pulse (SFQ pulse) phenomena and single biological neuron spike, which both have a similarity with the dynamics of the driven and damped pendulum. Indeed, with the designations for normalized frequency $\omega_{p}$ and damping parameter Γ:

(15) $$\begin{array}{c} \omega_{p}^{2}=\frac{2\pi I_{C}}{\Phi_{0}C},\;\Gamma^{2}=\frac{\Phi_{0}}{2\pi I_{C}{R_{N}}^{2}C}, \end{array}$$

the RCSJ model (10) becomes:

(16) $$\begin{array}{c} i_{x}=\frac{1}{\omega_{p}^{2}}\ddot{\delta_{x}}+\frac{\Gamma }{\omega_{p}}\dot{\delta_{x}}+\sin\delta_{x}, \end{array}$$

and $\omega_{p}$-time scaling (11) simplifies (16) to:

(17) $$\begin{array}{c} i_{x}=\ddot{\delta_{x}}+\Gamma \dot{\delta_{x}}+\sin\delta_{x}, \end{array}$$

which displays an analogy between the dynamics of a Josephson junction and a gravitational pendulum. In the generalized Equation (17), the state variable δ may represent both the Josephson phase and the angle of deflection of a pendulum. The model parameters can be set in such a way that for a time-dependent moment $i_{x}$, the pendulum will whirl only once and settle to the initial state. Such a whirling motion is utilized in a digital component from superconductive logic circuitry called a “DC-to-SFQ converter”. In [26], this schematic was simplified to obtain a circuit that behaves quite similarly to a biological neuron.

The original scheme contains two Josephson junctions with letter indices *p* and *c*, which stand for pulse and control, respectively. The control junction is located by the node where the bias current $I_{b}$ flows in. The signal of the neuron, which travels further to the synapse, is the voltage across the pulse junction $v_{p}=\dot{\phi_{p}}$. The pulse voltage $v_{p}$ simulates a polarizing ionic current $I_{Na+}$ of a biological neuron, and the control voltage $v_{c}$ simulates a hyperpolarizing ionic current $I_{K+}$. To increase the ability to control the throughput of the input channel, Schegolev et al. [33] replaced the control junction with a two-JJ superconducting interferometer. In a proposed 3-JJ-based neuron, the switching between all operating modes is possible by controlling only the bias current $I_{b}$ in a significantly larger range of parameters. Another benefit of this circuit is that all the junctions may be similar with respect to providing a working physical device.

**Figure 3:**
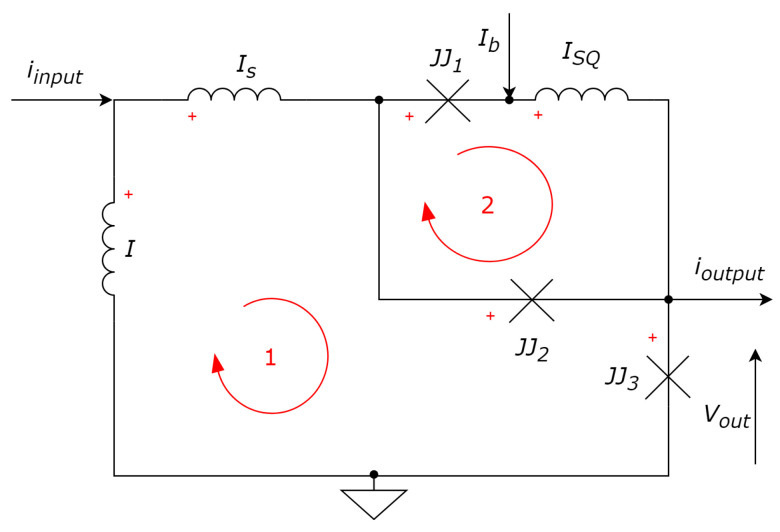
Circuit diagram of a modified spiking neuron based on 3 JJs from [33]. Red arrows denote loops and clarify the application of Kirchhoff’s law.

Let us consider the 3-JJ-based neuron model [33] in detail to understand how it can be described mathematically (see Figure 3).

Using the Kirchhoff’s law to calculate currents in nodes, one can obtain:


$$\left\{\begin{array}{cc} I_{in}=I_{s}+I_{L}; & (18a)\\ I_{s}=I_{JJ1}+I_{JJ2}; & (18b)\\ I_{JJ1}+I_{b}=I_{SQ}; & (18c)\\ I_{JJ2}+I_{SQ}=I_{out}+I_{JJ3}. & (18d) \end{array}\right.$$

According to Kirchhoff’s law for voltages, we obtain:

(19) $$\left\{\begin{array}{c} -V_{L}+V_{S}+V_{JJ2}+V_{JJ3}=0;\\ -V_{JJ2}+V_{JJ1}+V_{SQ}=0. \end{array}\right.$$

Let us recall the equation for inductances and Josephson law, and substitute it in (19):


$$\left\{\begin{array}{cc} -L{\dot{I}}_{L}+L_{S}{\dot{I}}_{S}\frac{\Phi_{0}}{2\pi }{\dot{\delta }}_{2}+\frac{\Phi_{0}}{2\pi }{\dot{\delta }}_{3}=0; & (20a)\\ -\frac{\Phi_{0}}{2\pi }{\dot{\delta }}_{2}+\frac{\Phi_{0}}{2\pi }{\dot{\delta }}_{1}+I_{SQ}=0. & (20b) \end{array}\right.$$

From (20b):

(21) $$I_{SQ}=-\frac{\Phi_{0}}{2\pi L_{SQ}}\cdot \left(\delta_{1}-\delta_{2}\right);$$

Substituted into (18c):

(22) $$I_{JJ1}=-I_{b}-\frac{\Phi_{0}}{2\pi L_{SQ}}\cdot \left(\delta_{1}-\delta_{2}\right).$$

From (18b) and (20a):

(23) $$\begin{array}{c} \left(I_{JJ1}+I_{JJ2}\right)\cdot L_{S}=L\cdot I_{L}-\frac{\Phi_{0}}{2\pi }\cdot \left(\delta_{2}+\delta_{3}\right) \end{array}$$

From (18a) and (18b), $I_{L}=I_{in}-I_{S}=I_{in}-I_{JJ1}-I_{JJ2}$, and thus:

(24) $$\begin{array}{c} \left(I_{JJ1}+I_{JJ2}\right)=\frac{L}{L_{S}}\cdot \left(I_{in}-I_{JJ1}-I_{JJ2}\right)-\frac{\Phi_{0}}{2\pi \cdot L_{S}}\cdot \left(\delta_{2}+\delta_{3}\right)\to \end{array}$$

(25) $$L_{S}I_{S}=L\cdot \left(I_{in}-I_{S}\right)-\frac{\Phi_{0}}{2\pi }\cdot \left(\delta_{2}+\delta_{3}\right)\to$$

(26) $$I_{S}\cdot \left(L_{S}+L\right)=I_{in}\cdot L-\frac{\Phi_{0}}{2\pi }\cdot \left(\delta_{2}+\delta_{3}\right).$$

Let us introduce the scaling coefficients and designations as follows.

(27) $$l_{x}=\frac{2\pi I_{C}L_{x}}{\Phi_{0}},\;\lambda =\frac{1}{l+l_{S}},\;i=\frac{I}{I_{C}}.$$

where $l_{x}\in \left\{l,\;l_{S},\;l_{SQ}\right\}$. Then:

(28) $$\begin{array}{cc} i_{S} & =l\cdot \lambda \cdot i_{in}-\lambda \cdot \left(\delta_{2}+\delta_{3}\right). \end{array}$$

Finally, after some substitutions:

(29) $$\left\{\begin{array}{c} i_{JJ1}=-i_{b}-\frac{1}{l_{SQ}}\cdot \left(\delta_{1}-\delta_{2}\right);\\ i_{JJ2}=i_{b}+\frac{1}{l_{SQ}}\cdot \left(\delta_{1}-\delta_{2}\right)+i_{in}\cdot \lambda \cdot l-\lambda \cdot \left(\delta_{1}+\delta_{2}\right);\\ i_{JJ3}=i_{b}+i_{in}\cdot \lambda \cdot l-\lambda \cdot \left(\delta_{1}+\delta_{2}\right)-i_{out}. \end{array}\right.$$

In the final ODE for a real system, the only difference from (17) is in the equation for $i_{JJ2}$, where the ratio of cross-sectional areas of the second and third Josephson junctions $\eta =A_{2}/A_{3}$ is considered:

$$i_{JJ2}=\eta \cdot \left({\ddot{\delta }}_{JJ2}+\Gamma {\dot{\delta }}_{JJ2}+\sin\delta_{JJ2}\right).$$

Numerical values for obtaining a realistic bursting mode of operation are as follows: $i_{b}=1.9$, l=5, $l_{s}=3.85$, $l_{SQ}=8.85$, λ=0.113, Γ=0.75, η=0.125. The neuron should be excited by input rectangular pulses with level $A_{input}=0.5$ and pulse duration $\tau =20t_{p}$.

### 2.3. Proposed Neuron-SQUID Model

**Figure 4:**
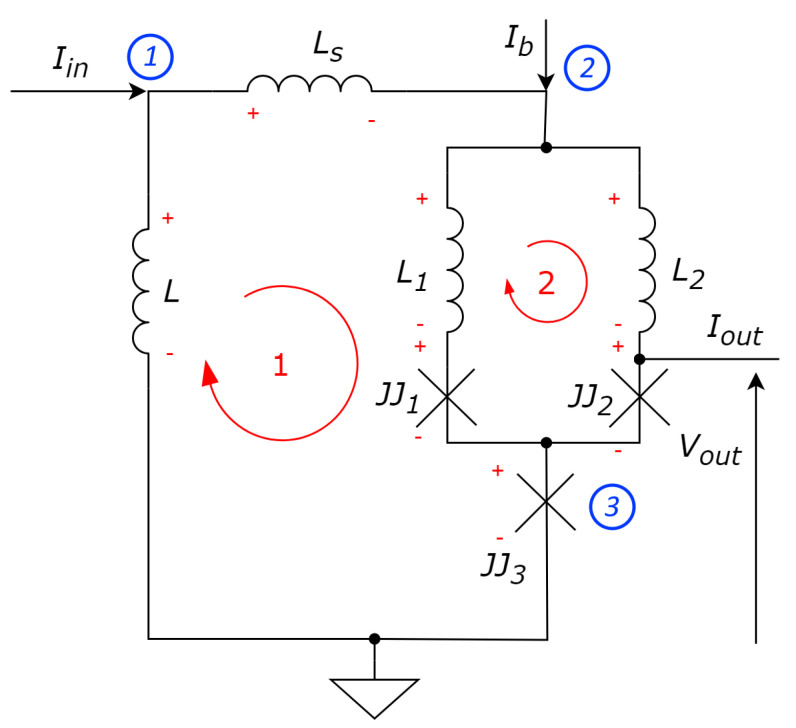
Proposed circuit design of the modified bio-inspired superconductive spiking neuron with integrated SQUID. Red arrows denote loops and blue numbers denote node indices in the equation system (30).

Figure 4 presents the schematic of the proposed device. Contrary to Schegolev’s model shown in Figure 3, our device has a DC SQUID (Figure 2) instead of the original superconducting interferometer.

Assume that inductances $L_{1}=L_{2}=\frac{L_{\Sigma }}{2}$ are the parts of the superconductive ring used to detect external flux $\phi_{e}\in N$ (discrete values of the quantized magnetic flux flowing through the ring). According to Kirchhoff’s law for currents in nodes, we obtain:


$$\left\{\begin{array}{cc} I_{in}=I_{s}+I_{L}; & (30a)\\ I_{s}+I_{b}=I_{JJ1}+I_{JJ2}; & (30b)\\ I_{JJ1}+I_{JJ2}=I_{JJ3}+I_{out}; & (30c) \end{array}\right.$$

According to Kirchhoff’s law for voltages:


$$\left\{\begin{array}{cc} -V_{L}+V_{S}+V_{L1}+V_{JJ2}+V_{JJ3}=0; & (31a)\\ -V_{JJ1}-V_{L_{1}}+V_{JJ2}+V_{L_{2}}=0. & (31b) \end{array}\right.$$

Compared with (8), it follows from (30b) and (31b):


$$\left\{\begin{array}{cc} V_{JJ1}=\frac{I_{b}+I_{s}}{2}+\frac{\Phi_{0}}{2\pi L_{\Sigma }}(\delta_{1}-\delta_{2}-2\pi \phi_{e}); & (32a)\\ V_{JJ2}=\frac{I_{b}+I_{s}}{2}-\frac{\Phi_{0}}{2\pi L_{\Sigma }}(\delta_{1}-\delta_{2}-2\pi \phi_{e}). & (32b) \end{array}\right.$$

From (30c):

(33) $$\begin{array}{c} -LI_{L}+L_{S}I_{S}+L_{1}I_{JJ1}+\frac{\Phi_{0}}{2\pi }\left(\delta_{1}+\delta_{3}\right)=0 \end{array}$$

From (30a), $I_{L}=I_{in}-I_{S}$, and thus:

(34) $$\begin{array}{c} -L\left(I_{in}-I_{S}\right)+L_{S}I_{S}=-\frac{L_{\Sigma }}{2}I_{JJ1}-\frac{\Phi_{0}}{2\pi }\left(\delta_{1}+\delta_{3}\right) \end{array}$$

Using designations (27), one can express the normalized current through the coil $L_{S}$, denoted as $i_{S}$, as follows:

(35) $$\begin{array}{c} i_{S}=l\cdot \lambda \cdot i_{in}-\frac{l_{\Sigma }}{2}\cdot \lambda \cdot i_{JJ1}-\lambda \cdot \left(\delta_{1}+\delta_{3}\right) \end{array}$$

Using another Kirchhoff’s law loop for calculating voltages, one may express $i_{S}$ as follows:

(36) $$\begin{array}{c} i_{S}=l\cdot \lambda \cdot i_{in}-\frac{l_{\Sigma }}{2}\cdot \lambda \cdot i_{JJ2}-\lambda \cdot \left(\delta_{2}+\delta_{3}\right) \end{array}$$

Substitute (35) into (32a):

(37) $$\begin{array}{c} \left(\frac{4+l_{\Sigma }\cdot \lambda }{2}\right)i_{JJ1}=i_{b}+l\cdot \lambda \cdot i_{in}-\frac{\lambda }{2}\cdot \left(\delta_{1}+\delta_{3}\right)+\frac{1}{l_{\Sigma }}\cdot \left(\delta_{1}-\delta_{2}-2\pi \phi_{e}\right) \end{array}$$

Denote $\lambda_{1}=2/\left(4+l_{\Sigma }\cdot \lambda \right)$. Furthermore, let $i_{out}=0$, so $i_{JJ3}=i_{JJ1}+i_{JJ2}$. Finally, we get:

(38) $$\left\{\begin{array}{c} i_{JJ1}=\lambda_{1}\cdot i_{b}+l\cdot \lambda \cdot \lambda_{1}\cdot i_{in}-\lambda \cdot \lambda_{1}\cdot \left(\delta_{1}+\delta_{3}\right)+\frac{2\lambda_{1}\cdot \left(\delta_{1}-\delta_{2}-2\pi \phi_{e}\right)}{l_{\Sigma }};\\ i_{JJ2}=\lambda_{1}\cdot i_{b}+l\cdot \lambda \cdot \lambda_{1}\cdot i_{in}-\lambda \cdot \lambda_{1}\cdot \left(\delta_{2}+\delta_{3}\right)-\frac{2\lambda_{1}\cdot \left(\delta_{1}-\delta_{2}-2\pi \phi_{e}\right)}{l_{\Sigma }};\\ i_{JJ3}=2\lambda_{1}\cdot i_{b}+2l\cdot \lambda \cdot \lambda_{1}\cdot i_{in}-\lambda \cdot \lambda_{1}\cdot \left(\delta_{1}+\delta_{2}+2\delta_{3}\right). \end{array}\right.$$

Thus, the RCSJ model of the JJs yields:

(39) $$\left\{\begin{array}{c} i_{JJ1}=\eta_{1}\cdot \left({\ddot{\delta }}_{1}+\Gamma {\dot{\delta }}_{1}+\sin\delta_{1}\right);\\ i_{JJ2}=\eta_{2}\cdot \left({\ddot{\delta }}_{2}+\Gamma {\dot{\delta }}_{2}+\sin\delta_{2}\right);\\ i_{JJ3}={\ddot{\delta }}_{3}+\Gamma {\dot{\delta }}_{3}+\sin\delta_{3}. \end{array}\right.$$

where the ratios $\eta_{1}=A_{1}/A_{3}$ and $\eta_{2}=A_{2}/A_{3}$ of cross-sectional areas of the first and third and and second and third Josephson junctions, respectively, should not be similar to provide the necessary asymmetry of DC SQUID and contribute to the generation of spikes. Merging (38) and (39), we obtain the differential equation describing neuron model dynamics.

To facilitate the reproducibility of this model, we provide the corresponding finite-difference scheme in Appendix A. This scheme is useful when analyzing dynamical regimes on the model in environments with difficulties in using high-level ODE solvers, e.g., in CUDA.

## 3. Results

### 3.1. Experimental Setup

We used NI LabVIEW 2022 Q3 as a simulation environment for investigating the waveforms. To increase performance, two-parameter diagrams were constructed using proprietary software written in C++ Nvidia CUDA Compiler (NVCC) v. 9.0 by Nvidia corporation (San Tomas Expressway, Santa Clara, CA 95051, USA) to run on GPU supporting CUDA platform. The utilized computer hardware is as follows:

- Intel Core i9 12,900k CPU;
- Nvidia GeForce RTX 4090 GPU;
- 64 GB DDR4 RAM;
- 2 TB SSD storage device.

Considering recent developments in numerical approaches to nonlinear and chaotic problems [50], we suggest that the semi-implicit CD numerical integration method [51,52] would yield better correspondence to the reference and better reflect the dynamical features of the continuous system, also maintaining acceptable performance for a multi-parametric examination. To investigate the properties of the flux-sensitive 3-JJ neuron, we simulated it using the semi-implicit CD method with time step $h=0.01t_{p}$ ($\omega_{p}$-scaled time). If not specified otherwise, system parameters in our study are as follows: $i_{b}=1$, l=3, λ=0.5, $l_{\Sigma }=8$, $\eta_{i}=1$, Γ=2, the amplitude of $i_{in}$ is $A_{input}=1$, pulse duration $\tau_{d}=20t_{p}$ and pulse period $\tau_{p}=240t_{p}$.

### 3.2. Dynamical Modes of the Neuron-SQUID

In Ref. [33], the authors distinguish the following types of oscillation modes or regimes:

1. The dead mode, which corresponds to a neuron that does not respond to input stimuli—this mode can also be characterized by a very small amplitude;
2. The injury mode, where only some of the input stimuli generate a spike in response;
3. The regular mode, where a standard input stimulus generates a response spike;
4. The bursting mode, where a standard input stimulus leads to the generation of a sequence of spikes, so-called bursts;
5. The nonbiological mode, where the signal is highly biased to the positive region and the spikes have small amplitude and high frequency. Such an output signal is not found in biological neurons.

**Figure 5:**
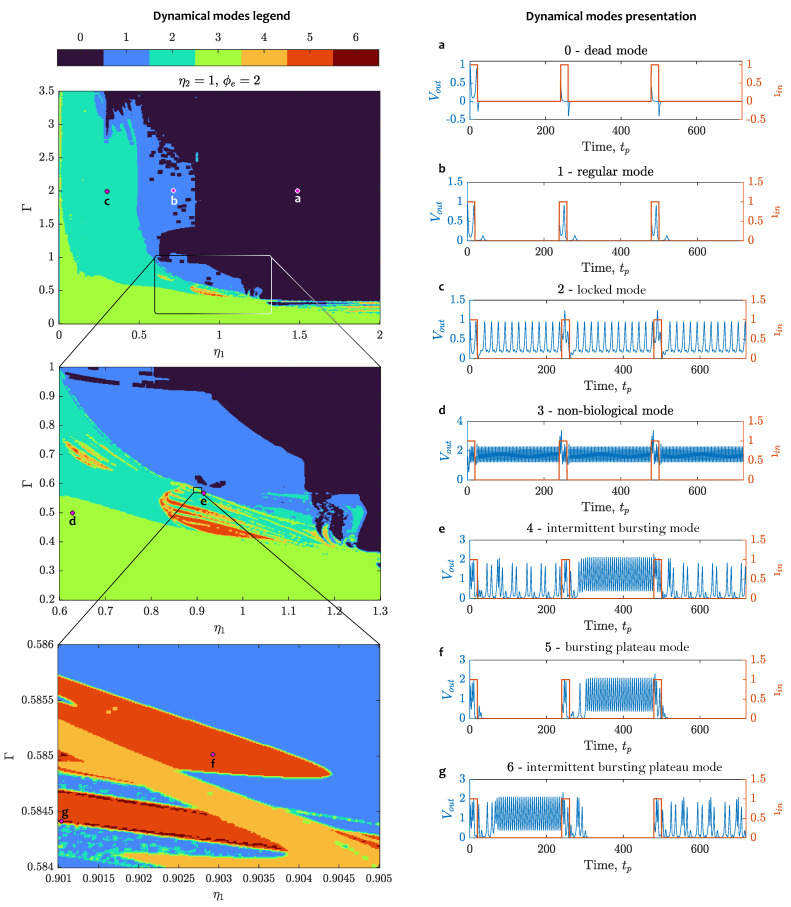
Neuron-SQUID operating modes for damping parameter Γ and geometric factor $\eta_{1}$: (**a**) dead mode; (**b**) regular mode; (**c**) locked spiking mode; (**d**) nonbiological mode; (**e**) intermittent bursting mode; (**f**) bursting plateau mode; (**g**) intermittent bursting plateau mode. Violet diamonds on the parameter plane represent pairs of the parameter values Γ and $\eta_{1}$ for the corresponding modes, while $\eta_{2}=1$ and $\phi_{e}=2$. Other simulation parameters are listed in Section 3.1.

To investigate the dependence between the operational modes of the proposed 3-JJ neuron and system parameters, we plotted diagrams for two-dimensional dynamics, as shown in Figure 5. In [33], the authors state that for JJ-based neurons, all modes can be induced by varying the geometric factor η and damping parameter Γ. In our study, we introduced two geometric factors, $\eta_{1}$ and $\eta_{2}$. In the proposed neuron model, we discovered some modes that are similar to [33], but also found that the proposed flux-sensitive topology may demonstrate richer dynamics. Visualization of the dynamical behavior in the presence of a weak magnetic flux can be seen in Figure 5. One may see that in its parameter plane, the neuron dynamics reveal its fractal structure inherent to nonlinear systems.

To characterize the observed dynamics, we introduce the following classification of modes:

1. Dead mode, or weak dynamical response. In this mode, spikes with low amplitude follow the input strobe, getting wider and changing their phase with the increase of $\phi_{e}$.
2. Regular mode, where a standard input stimulus generates a response spike or a series of nonuniform spikes. Oscillations in the neuron fade out and do not appear until the next stimulus.
3. Locked mode [53,54]. In this mode, oscillations in the neuron appear either due to the input stimulus or autonomously and represent continuous chain spikes following each other with equal interspike intervals, which decreases with the growth of the external magnetic flux $\phi_{e}$. At some combinations of parameters, single spikes are replaced by short bursts (duplets and triplets of spikes), the regularity of oscillations disappears and the system goes into chaos. Nevertheless, the total spiking rate is approximately preserved.
4. Nonbiological mode. Any type of behavior that has no counterpart or analog in biological neurons. One type of nonbiological behavior is characterized by very fast spiking with low amplitude around positively biased value $V_{out}>>0$. Another observed type of nonbiological behavior is chaotic oscillations resembling sinusoidal beats, which occur at low values of Γ or $\eta_{i}$.
5. Intermittent bursting. In this mode, instead of single spikes or spike groups, the neuron starts generating a continuous sequence of very short spikes by the action of an input stimulus. The next stimulus leads the neuron from bursting back to spiking.
6. Bursting plateau mode [55]. Due to the occurrence of a plateau potential inside a neuron, under the action of the input stimulus, the neuron gets excited and maintains firing. The next impulse turns the neuron off.
7. Intermittent bursting plateau mode—a combination of the two aforementioned modes. Under the action of an input stimulus, a neuron may stop spiking, or on the contrary, maintain bursting. This mode is rather difficult to detect; nevertheless, it is observed in different regions of parameter values.

**Figure 6:**
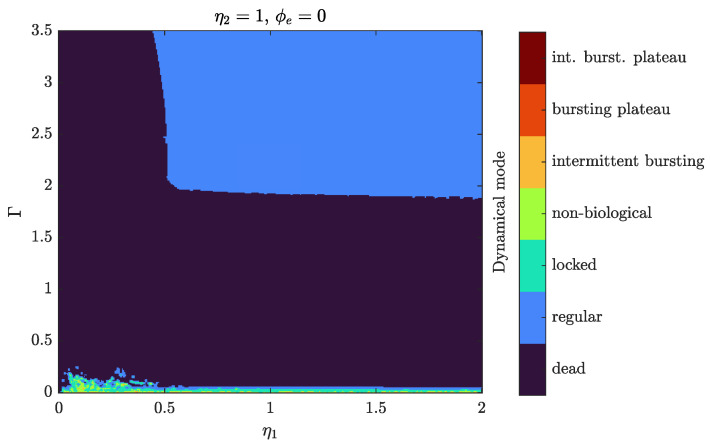
Neuron-SQUID operating modes without external flux. Near the value of damping parameter Γ=0, the nonbiological mode predominantly presents.

It should be noted that the mentioned diversity of neuron-SQUID modes is observed in the presence of an external magnetic flux. When the external magnetic flux is absent, the dynamics of the neuron are quite poor (see Figure 6). In particular, no bursting modes can be observed without flux and even locked spiking mode is questionable to detect and looks similar to nonbiological modes.

**Figure 7:**
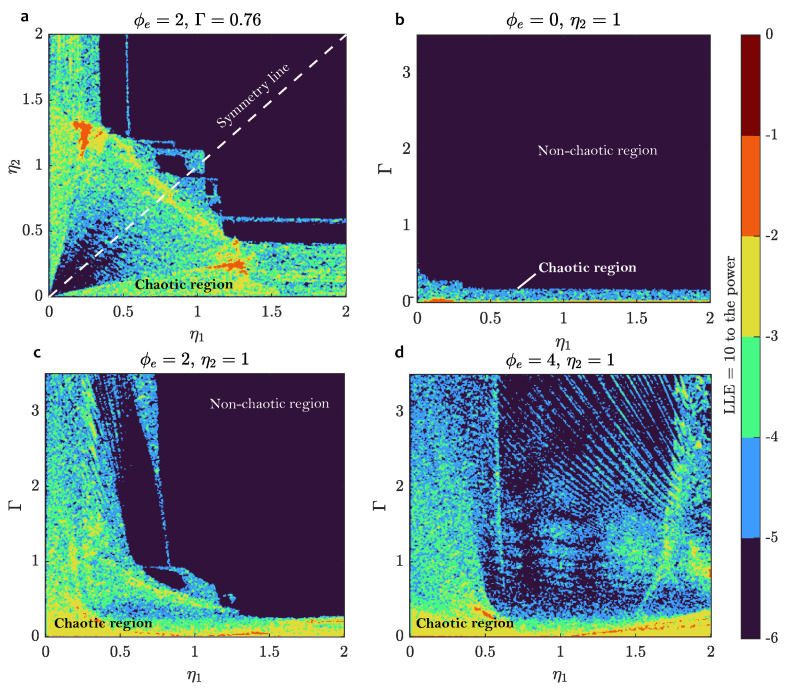
Neuron-SQUID LLE analysis: (**a**) in $\eta_{1}$-$\eta_{2}$ plane; (**b**–**d**) in Γ-$\eta_{1}$ plane, for different values of $\phi_{e}$. The asymmetry of Josephson contacts in the SQUID ring contributes to chaotic dynamics, as well as the increase of an external magnetic flux.

Assuming that the complexity of the neuron dynamics is connected to chaotic behavior, we calculated two-dimensional diagrams of the largest Lyapunov exponents (LLE) (see Figure 7). Using this type of analysis, we found that the asymmetrical DC SQUID ring ($\eta_{1}\neq \eta_{2}$) is important and provides rich dynamics of the neuron (see Figure 7a). Near the symmetry line $\eta_{1}=\eta_{2}$, LLE is mostly negative or has a very small positive value, while at some nonsymmetrical pair LLE >>0, which results in complex behavior, e.g., intermittent bursting plateau mode.

Speaking of the influence of external flux, its increase causes the neuron to switch from regular mode to locked spiking, and then to nonbiological mode. Usually, the nonbiological mode is chaotic. Bursting modes are found in between locked and nonbiological modes.

### 3.3. Quantification of Neuron-SQUID Reaction to the External Flux

**Figure 8:**
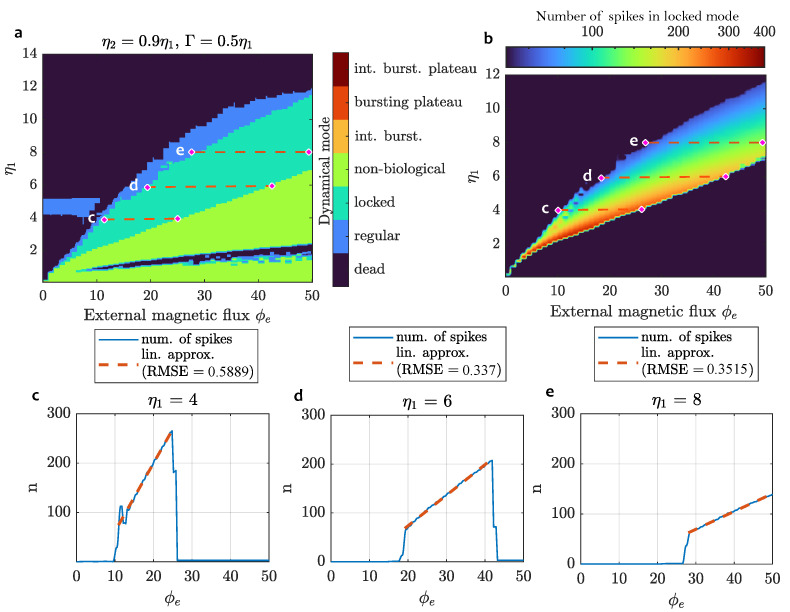
Evaluation of neuron-SQUID performance as a magnetic flux sensor: (**a**) neuron-SQUID operating modes; (**b**) visualization of a number of spikes count during time $t=1440t_{p}$ (six periods of current stimulation) when neuron behaves in the locked mode; (**c**–**e**) plots for a number of spikes and corresponding linear approximations at different values of system parameters.

To visualize the dynamics of the spiking behavior, we plotted the distribution of spikes as a function of the input parameter—external magnetic flux $\phi_{e}$. The dynamics for different values of the parameter η are shown in Figure 8. Note that with the change of $\eta_{1}$ along the *Y* axis, the parameters $\eta_{2}$ and Γ of the neuron under study also change. This allows one to better adapt it to a given range of magnetic flux variation. Speaking of several types of complex behavior presented in Figure 5, they can be observed on the thin edge between the locked and nonbiological modes and require different ratios between the three main parameters to be more noticeable at this scale.

The experiments show that the system demonstrates activity in a wide range of magnetic flux $\phi_{e}$ values when the parameters are changed, from a few quanta to tens and even hundreds (not shown). Thus, by choosing the required geometrical characteristics of the sensor at the fabrication stage, it is possible to set its sensitivity according to the task. When $\eta_{i}<1$, the sensor is capable of detecting a single flux quantum.

A remarkable property of the discovered dependency between the number of spikes in the locked mode and the magnetic flux is its high linearity. This makes it possible to design magnetic flux sensors based on the proposed 3-JJ neural structure with a simple interpretation of the output. In Table 1, the expressions for linear approximation of dependencies $\phi_{e}\left(n\right)$ from Figure 8 are presented.

Note that the choice of neuron-SQUID parameters for the study presented in this section is grounded by the sensitivity of the neuron. In addition to geometrical parameters $\eta_{i}$ and Γ for an external magnetic flux $\phi_{e}$, we could also characterize the influence of the inductance parameters *l*, $l_{\Sigma }$ and λ, input bias current $i_{b}$, and input stimuli *A* and $\tau_{i}$. However, we did not find any significant nonlinear effects caused by these parameters on the dynamics of the neuron.

## 4. Conclusions

In this study, we considered a novel neuron model based on Josephson junctions (JJs), which is proven to be sensitive to external magnetic flux. The investigated circuit consists of several elements: two inductances, a separate Josephson junction, and a DC SQUID ring, which also includes a couple of JJs. This ring is responsible for the neuron’s sensitivity to magnetic flux. As the key results of our study, we made the following findings.

1. The performance and key nonlinear properties of the proposed topology were experimentally confirmed by numerical simulation. We discovered that the input pulse current and external flux provide the device functionality that resembles the activity of the biological neuron.
2. The system exhibits a wide range of dynamical modes inherent to neurons, including regular spiking, locked spiking, intermittent bursting, plateau bursting, and the combination of the two latter. Complex modes of neuron behavior are explained by the emergence of chaos, which was confirmed by the largest Lyapunov exponent calculation.
3. The sensitivity of the proposed neuron model to an external magnetic field can be controlled by selecting its physical parameters, such as the ratio of areas of JJs in the DC SQUID and area of the free-standing JJ (so-called geometric factors $\eta_{i}$), and the damping parameter Γ. For different values of these parameters, the neuron demonstrates sensitivity in various ranges of magnetic flux, from single quantum to tens and hundreds of quants. Moreover, we found that for a certain range of $\phi_{e}$, the neuron firing rate is linearly proportional to the magnetic flux $\phi_{e}$ when the neuron operates in the locked spiking mode. The theoretical precision of such a sensor in terms of root-mean-square error is less than a single quantum. For example, with main parameters values $\eta_{1}=6$, $\eta_{2}=5.4$ and Γ=3, the RMSE=0.34 in the range of 23 quanta, from $\phi_{e}=19$ to $\phi_{e}=42$.

Following the in-memory computation paradigm which can be implemented using JJ-based neuromorphic hardware, the outputs of the proposed neurons may drive a spiking neural network, and the magnetic field values encoded in spiking dynamics could be naturally processed and recognized by this network. Simulation of such a system, as well as verification of the physical implementability of the proposed device, are the aims of further research. However, we must note that processing of incoming data by the capabilities of superconductor-based neural network computing remains a challenge, since it requires a cryosystem with appropriate shielding to eliminate adverse external influence.

## Figures and Tables

**Table 1:** Numerical evaluations of neuron-SQUID performance as a magnetic flux sensor, with a linear approximation of spiking rate response in the locked spiking mode of operation.

System Parameters	$\phi_{e}\left(n\right)$	RMSE	$\phi_{e}$ Range	$\phi_{e}$ Range Width
$\eta_{1}$ = 4, $\eta_{2}$ = 3.6, Γ = 2	0.0726n+5.4793	0.59	[11; 25]	14
$\eta_{1}$ = 6, $\eta_{2}$ = 5.4, Γ = 3	0.1595n+8.2602	0.34	[19; 42]	23
$\eta_{1}$ = 8, $\eta_{2}$ = 7.2, Γ = 4	0.2811n+10.4862	0.35	[28; 58]	30

## Data Availability

No new data were created or analyzed in this study.

## Appendix A. Finite-Difference Scheme of the Neuron-SQUID Model

By obtaining the equations for the circuit under investigation, we can finally build a finite difference scheme of equations for performing numerical experiments. In our study, we use the CD integration method [51]. It should be noted that in its original form, the scheme is a nonautonomous system of ordinary differential equations of the second order, which can be easily converted into an autonomous system of ordinary differential equations of the first order by replacing variables and adding another equation for calculating time:

(A1) $${\dot{\delta }}_{1}=y_{1},\;{\dot{\delta }}_{2}=y_{2},\;{\dot{\delta }}_{3}=y_{3}\to {\ddot{\delta }}_{1}={\dot{y}}_{1},\;{\ddot{\delta }}_{2}={\dot{y}}_{2},\;{\ddot{\delta }}_{3}={\dot{y}}_{3}.$$

Using the change of variables from (A1) and substituting them into (39), we obtain the following system of ordinary differential equations:

(A2) $$\left\{\begin{array}{c} {\dot{\delta }}_{1}=y_{1};\\ {\dot{\delta }}_{2}=y_{2};\\ {\dot{\delta }}_{3}=y_{3};\\ {\dot{y}}_{1}=\frac{i_{JJ1}\left(\delta_{1},\delta_{2},\delta_{3},i_{in}\right)}{\eta_{1}}-\Gamma y_{1}-\sin\delta_{1};\\ {\dot{y}}_{2}=\frac{i_{JJ2}\left(\delta_{1},\delta_{2},\delta_{3},i_{in}\right)}{\eta_{2}}-\Gamma y_{2}-\sin\delta_{2};\\ {\dot{y}}_{3}=i_{JJ3}\left(\delta_{1},\delta_{2},\delta_{3},i_{in}\right)-\Gamma y_{3}-\sin\delta_{3}. \end{array}\right.$$

where:

(A3) $$\begin{array}{c} \begin{array}{cc} i_{JJ1}\left(\delta_{1},\delta_{2},\delta_{3},i_{in}\right) & =\lambda_{1}\cdot \left(i_{b}+l\cdot \lambda \cdot i_{in}-\lambda \cdot \left(\delta_{1}+\delta_{3}\right)+\frac{2(\delta_{1}-\delta_{2}-2\pi \phi_{e})}{l_{\Sigma }}\right);\\ i_{JJ2}\left(\delta_{1},\delta_{2},\delta_{3},i_{in}\right) & =\lambda_{1}\cdot \left(i_{b}+l\cdot \lambda \cdot i_{in}-\lambda \cdot \left(\delta_{2}+\delta_{3}\right)-\frac{2(\delta_{1}-\delta_{2}-2\pi \phi_{e})}{l_{\Sigma }}\right);\\ i_{JJ3}\left(\delta_{1},\delta_{2},\delta_{3},i_{in}\right) & =\lambda_{1}\cdot \left(2i_{b}+2l\cdot \lambda \cdot i_{in}-\lambda \cdot \left(\delta_{1}+\delta_{2}+2\delta_{3}\right)\right). \end{array} \end{array}$$

Method CD is a composition of a pair of basic adjoint D-methods. The first adjoint method is fully explicit, and the second adjoint method contains implicitness in the diagonal elements of the system matrix. A family of adjoint semi-implicit methods with variable symmetry appears:

(A4) $$\Psi_{h,s}=\Phi_{h_{1}}\circ \Phi_{h_{2}}^{*};$$

where:

(A5) $$\left\{\begin{array}{c} h_{1}=h\cdot s;\\ h_{2}=h\cdot \left(1-s\right). \end{array}\right.$$

Let us apply the CD method for the system (A3). Building the explicit half is quite straightforward and it can be written as follows:

(A6) $$\Phi_{h_{1}}=\left\{\begin{array}{c} \delta_{1,n+s}=\delta_{1,n}+h_{1}\cdot y_{1,n};\\ \delta_{2,n+s}=\delta_{2,n}+h_{1}\cdot y_{2,n};\\ \delta_{3,n+s}=\delta_{3,n}+h_{1}\cdot y_{3,n};\\ y_{1,n+s}=y_{1,n}+h_{1}\cdot \left(\frac{i_{JJ1}\left(\delta_{1,n+s},\delta_{2,n+s},\delta_{3,n+s},i_{in}\right)}{\eta_{1}}-\Gamma y_{1,n}-\sin\delta_{1,n+s}\right);\\ y_{2,n+s}=y_{2,n}+h_{1}\cdot \left(\frac{i_{JJ2}\left(\delta_{1,n+s},\delta_{2,n+s},\delta_{3,n+s},i_{in}\right)}{\eta_{2}}-\Gamma y_{2,n}-\sin\delta_{2,n+s}\right);\\ y_{3,n+s}=y_{3,n}+h_{1}\cdot \left(i_{JJ3}\left(\delta_{1,n+s},\delta_{2,n+s},\delta_{3,n+s},i_{in}\right)-\Gamma y_{3,n}-\sin\delta_{3,n+s}\right). \end{array}\right.$$

Now let us build the one with the implicitness in the diagonal elements of the system matrix:

(A7) $$\Phi_{h_{2}}^{*}=\left\{\begin{array}{c} y_{3,n+1}=y_{3,n+s}+h_{2}\cdot \left(i_{JJ3}\left(\delta_{1,n+s},\delta_{2,n+s},\delta_{3,n+s},i_{in}\right)-\Gamma y_{3,n+1}-\sin\delta_{3,n+s}\right).\\ y_{2,n+1}=y_{2,n+s}+h_{2}\cdot \left(\frac{i_{JJ2}\left(\delta_{1,n+s},\delta_{2,n+s},\delta_{3,n+s},i_{in}\right)}{\eta_{2}}-\Gamma y_{2,n+1}-\sin\delta_{2,n+s}\right);\\ y_{1,n+1}=y_{1,n+s}+h_{2}\cdot \left(\frac{i_{JJ1}\left(\delta_{1,n+s},\delta_{2,n+s},\delta_{3,n+s},i_{in}\right)}{\eta_{1}}-\Gamma y_{1,n+1}-\sin\delta_{1,n+s}\right);\\ \delta_{3,n+1}=\delta_{3,n+s}+h_{2}\cdot y_{3,n+1};\\ \delta_{2,n+1}=\delta_{2,n+s}+h_{2}\cdot y_{2,n+1};\\ \delta_{1,n+1}=\delta_{1,n+s}+h_{2}\cdot y_{1,n+1}, \end{array}\right.$$

where the implicitness can be resolved analytically:

(A8) $$\Phi_{h_{2}}^{*}=\left\{\begin{array}{c} y_{3,n+1}=\frac{y_{3,n+s}+h_{2}\cdot \left(i_{JJ3}\left(\delta_{1,n+s},\delta_{2,n+s},\delta_{3,n+s},i_{in}\right)-\sin\delta_{3,n+s}\right)}{1+h_{2}\Gamma };\\ y_{2,n+1}=\frac{y_{2,n+s}+h_{2}\cdot \left(\frac{i_{JJ2}\left(\delta_{1,n+s},\delta_{2,n+s},\delta_{3,n+s},i_{in}\right)}{\eta_{2}}-\sin\delta_{2,n+s}\right)}{1+h_{2}\Gamma };\\ y_{1,n+1}=\frac{y_{1,n+s}+h_{2}\cdot \left(\frac{i_{JJ1}\left(\delta_{1,n+s},\delta_{2,n+s},\delta_{3,n+s},i_{in}\right)}{\eta_{1}}-\sin\delta_{1,n+s}\right)}{1+h_{2}\Gamma };\\ \delta_{3,n+1}=\delta_{3,n+s}+h_{2}\cdot y_{3,n+1};\\ \delta_{2,n+1}=\delta_{2,n+s}+h_{2}\cdot y_{2,n+1};\\ \delta_{1,n+1}=\delta_{1,n+s}+h_{2}\cdot y_{1,n+1}. \end{array}\right.$$

Briefly, the advantages of the proposed integration scheme are as follows: it has the second order of algebraic accuracy, provides higher stability in comparison with explicit methods such as Runge–Kutta, but it is still similarly computationally efficient. For more details, refer to [50].
